# Antimicrobial use, residues and resistance in fish production in Africa: systematic review and meta-analysis

**DOI:** 10.1186/s12917-024-04158-w

**Published:** 2024-07-10

**Authors:** Frédéric Moffo, Mohamed Moustapha Fokom Ndebé, Mildred Naku Tangu, Ranyl Nguena Guefack Noumedem, Julius Awah-Ndukum, Mohamed Moctar Mouliom Mouiche

**Affiliations:** 1 Department of Pharmacy, Pharmacology and Toxicology, School of Veterinary Medicine and Sciences, University of Ngaoundéré, Ngaoundéré, Cameroon; 2 Department of Animal Science, Faculty of Agronomy and Agricultural Sciences, Laboratory of Animal Physiology and Health, University of Dschang, Dschang, Cameroon; 3 Institute of Agricultural Research for Development, Bangangté Polyvalent Station, Bangangté, Cameroon; 4 National Veterinary Laboratory (LANAVET), Garoua, Cameroon; 5 Department of Animal Production Technology, College of Technology, University of Bamenda, Bambili, Cameroon; 6 One Health Innovative Solutions (OHIS) Research Unit, Ngaoundéré, Cameroon

**Keywords:** Antimicrobial use, Antimicrobial residue, Antimicrobial resistance, Multidrug resistance, Systematic review, Meta-analysis, Aquaculture, Africa

## Abstract

In low- and middle-income countries, data on antimicrobial use (AMU) and antimicrobial resistance (AMR) in aquaculture are scarce. Therefore, summarizing documented data on AMU, antimicrobial residue (AR), and AMR in aquaculture in Africa is key to understanding the risk to public health. Google Scholar, PubMed, African Journals online, and Medline were searched for articles published in English and French following the PRISMA guidelines. A structured search string was used with strict inclusion and exclusion criteria to retrieve and screen the articles. The pooled prevalence and 95% confidence intervals were calculated for each pathogen–antimicrobial pair using random effects models. Among the 113 full-text articles reviewed, 41 met the eligibility criteria. The majority of the articles reported AMR (35; 85.4%), while a few were on AMU (3; 7.3%) and AR (3; 7.3%) in fish. The articles originated from West Africa (23; 56.1%), North Africa (8; 19.7%), and East Africa (7; 17.1%). Concerning the antimicrobial agents used in fish farming, tetracycline was the most common antimicrobial class used, which justified the high prevalence of residues (up to 56.7%) observed in fish. For AMR, a total of 69 antimicrobial agents were tested against 24 types of bacteria isolated. Bacteria were resistant to all classes of antimicrobial agents and exhibited high levels of multidrug resistance. *Escherichia coli*, *Salmonella* spp., and *Staphylococcus* spp. were reported in 16, 10, and 8 studies, respectively, with multidrug resistance rates of 43.1% [95% CI (32.0–55.0)], 40.3% [95% CI (24.1–58.1)] and 31.3% [95% CI (17.5–49.4)], respectively. This review highlights the high multidrug resistance rate of bacteria from aquaculture to commonly used antimicrobial agents, such as tetracycline, ampicillin, cotrimoxazole, gentamicin, and amoxicillin, in Africa. These findings also highlighted the lack of data on AMU and residue in the aquaculture sector, and additional efforts should be made to fill these gaps and mitigate the burden of AMR on public health in Africa.

## Background

Aquaculture is a rapidly growing livestock production sector with an expected increase of 62% in 2030, and it represents one of the most sustainable and economical sources of protein for humans [1]. It provides approximately 15% of the animal protein needs of more than three billion people worldwide [2]. However, infectious diseases seriously threaten aquaculture production and the livelihoods of many households [3], and fish farmers usually use antimicrobial agents for the prevention and control of diseases and as growth promoters [4]. However, antimicrobials that are not efficiently metabolized by fish are eliminated through urine and feces [3]. Additionally, chemical substances such as disinfectants and biocides used to ensure good water quality [5], together with ARs from integrated production systems, may contribute to the selection, emergence, and spread of drug-resistant pathogens, which pose serious threats to public health [4, 6]. Research has indicated that the use of antimicrobials as growth promoters in agriculture is associated with the emergence of resistant foodborne pathogens, which are relatively risky to human, animal, and environmental health [7]. In most African countries, the choice of antimicrobial agent is not usually based on knowledge of bacterial susceptibility tests [8].

The inappropriate use of antimicrobials has accelerated AMR emergence at animal, human and environmental interfaces [9–11]. In various low- and middle-income countries, published data on AMR are more frequently observed in animal and human compartments than in environmental compartments. However, the scarcity of data might hamper efforts to fight AMR from the environment and mainly from aquaculture to human and animal health [2]. Summarized available data are essential for the development of local and regional treatment guidelines, with an emphasis on the need for sustainable efforts by stakeholders for the coordination and harmonization of competencies against the emergence of AMR [8]. Therefore, this study was carried out to systematically analyze validated information on AMU, ARs, and AMR emergence in fish production systems in Africa.

## Methods

### Search strategy

This systematic review was performed following the PRISMA (Preferred Reporting Items for Systematic Reviews and Meta-Analysis) guidelines [12]. The PubMed, Google Scholar, and African Journal Online databases were used to search for articles published in English and French on AMU, AR, and AMR in Africa. No limit on publication date was set. The literature search started from November 2020 to August 2021. The reference lists of relevant articles were checked for additional titles for inclusion in the review. The free text was obtained by contacting the authors directly. Additionally, attempts were made to contact the authors to obtain inaccessible abstracts and full texts from the included studies. Boolean operators (AND/OR/NOT) and predefined search terms of relevant studies conducted in African countries in aquaculture and related production sectors were adopted [8, 13]. The following keywords were used: ‘antimicrobial use’, ‘antibiotic use’, ‘chemical use’, ‘antibiotic residue’, ‘antimicrobial residue’, ‘chemical residue’, ‘antimicrobial resistance’, ‘antibiotic resistance’, ‘chemical resistance’, ‘aquaculture’, ‘fish farm’, ‘fish’, ‘shellfish’, ‘shrimp’, ‘Africa’, and ‘specific African countries.

### Inclusion and exclusion criteria

The procedure for the inclusion and exclusion of articles in the systematic review and meta-analysis was similar to that described by Mouiche et al. [8]. Briefly, full-text articles published on AMU, AM, and the prevalence of AMR among bacteria isolated in aquaculture or natural aquatic milieu or in association with other food items in African countries were used in the review. After removing duplications and retracted citations in Zotero, the citations were uploaded to Rayyan software for screening. First, the selection process consisted of title and abstract screening. To increase consistency among reviewers, a calibration exercise was carried out on 10 randomly selected articles to enable discussion and resolve disagreements before the full-text selection process. Two authors (MMFN and FM) independently reviewed the publications to determine eligibility. When there was doubt about the decision, this was resolved by consensus or third-party consultation (MMMM and JAN) when consensus could not be reached. Publications that described aquatic subjects or aquatic populations studied or types of aquatic environmental samples, bacteria isolated, specific laboratory methods, antimicrobial sensitivity patterns, and antimicrobial tests were considered and included in the study. Articles obtained through the use of predefined search terms (which were translated to search articles written in French) to identify relevant literature were included. Studies on mycobacteriosis and outbreak disease were not included. Studies reporting aggregated data, such as studies in which resistance rates were aggregated in a large category, were excluded. Additionally, articles identified through a literature search that reported AMR in aquaculture and aquatic environments but that did not report prevalence data were not included in the meta-analysis.

### Data extraction

The data were extracted from individual studies using a form and database developed for this review in Microsoft Excel 2013. The data extraction was independently performed by two coauthors (MMFN and FM), while MNT and RNGN conducted the datachecking of the included papers. When there was a confrontation of the data set, third-party (MMMM and JAN) consultation was performed for validation. Articles that met the inclusion criteria and reported AMR data in aquaculture production and aquatic environments were included in the meta-analysis. The extracted information included article information: first author, year of publication, duration of study and country, study design (cross-sectional or longitudinal study), type of aquaculture production or aquatic products (type and species of aquatic species, processed and unprocessed), sampling point and origin of aqua-product, aquatic environment (farm, natural, market for aquatic products), and type of sample (fluids, gastrointestinal content, tissue, organs). Qualitative and quantitative data on AMU (type of antimicrobial agent, frequency and indication of usage), AR (antimicrobial agents investigated, quantity, prevalence), and AMR (number of strains tested for AMR, number of resistant strains, antimicrobial panels tested) as well as laboratory procedures and bacteria investigated were also taken into consideration.

A quality assessment of the articles was performed to evaluate the reliability of the studies using a modified version of a critical appraisal tool developed for use in systematic reviews addressing questions of prevalence [14]. Each publication was assessed using 5 specific questions: (1) If the data included study period, sample type, and study zone? (2) Were the study subjects and setting described in detail? (3) Was the data analysis conducted with sufficient coverage of the identified germ? (4) Were the objectives and standard criteria used to measure the condition? (5) Was the condition measured reliably? Responses to each of the five questions were coded as yes (Y), no (N), or unclear (U) and categorized into three groups. Articles that answered “yes” to ≥ 80% of the items were classified as high quality (H), articles that answered “yes” to 60%—< 80% of the items were considered medium quality (M), and articles that answered “yes” to less than 60% of the items were considered low quality (L). Articles that scored high quality (H) or medium quality (M) were included in the review.

### Data analysis

Descriptive statistics were used to summarize the characteristics of the articles included in the review and the AMU and AR data from the fish. For AMR, the point estimate prevalence and 95% confidence interval (CI) of each pathogen–antimicrobial pair were pooled using a random effects model. Resistance rates were pooled if at least four studies reported on a specific bacterium-antimicrobial combination. Random effects meta-analysis was also used to calculate the overall proportion of pathogen–multidrug resistance pairs. If not defined by the study, resistance to three or more antimicrobial classes, frequently used in primary reports, was considered multidrug resistance (MDR) [15].

### Subgroup analysis was performed according to the African region

Heterogeneity across the studies was assessed using the Cochrane Q statistic (significant at *p* < 0.10) and was quantified with the I^2^ statistic [13, 16]. Sensitivity analysis was performed to evaluate the influence of individual studies on the final effect. The Begg rank correlation [17] and Egger regression asymmetry test [18] were used to examine publication bias. If publication bias was confirmed, a trim-and-fill method developed by Duval and Tweedie [19] was used to adjust for the bias. The funnel plot was replicated with their “missing” counterparts around the adjusted summary estimate. If, after a detailed investigation, there was no obvious cause for the heterogeneity, the data were analyzed with a more conservative statistical method. Random effects analysis attempts to account for the distribution of effects and provides a more conservative estimate of the effect [16, 20]. A *p v*alue of 0.05 was considered to indicate statistical significance, except for the test of heterogeneity. The data were analyzed using Comprehensive Meta-Analysis Software (Biostat, Inc., New Jersey) Version 3.0 for Windows.

## Results

**Fig. 1:**
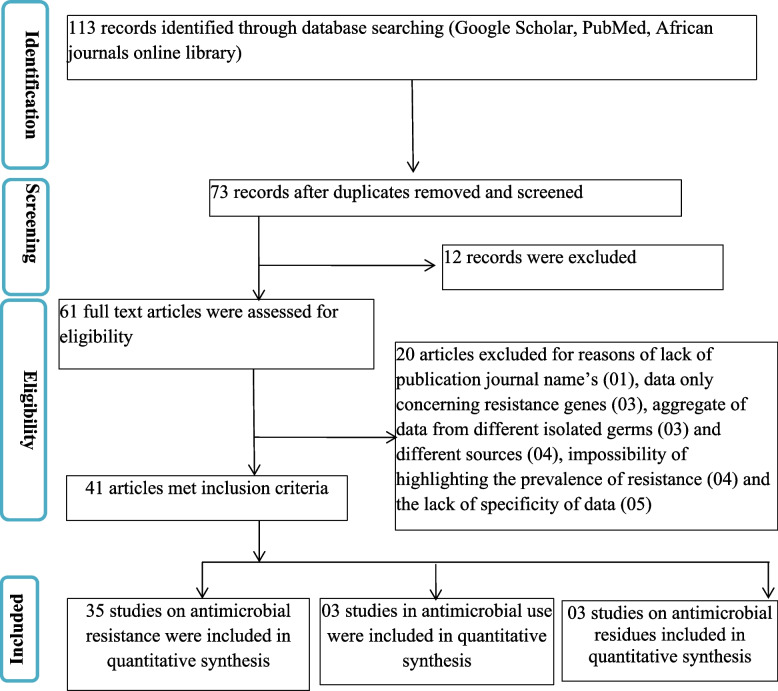
PRISMA flow chart illustrating the study selection process for antimicrobial use, antimicrobial residues and antimicrobial resistance in Africa

**Fig. 2:**
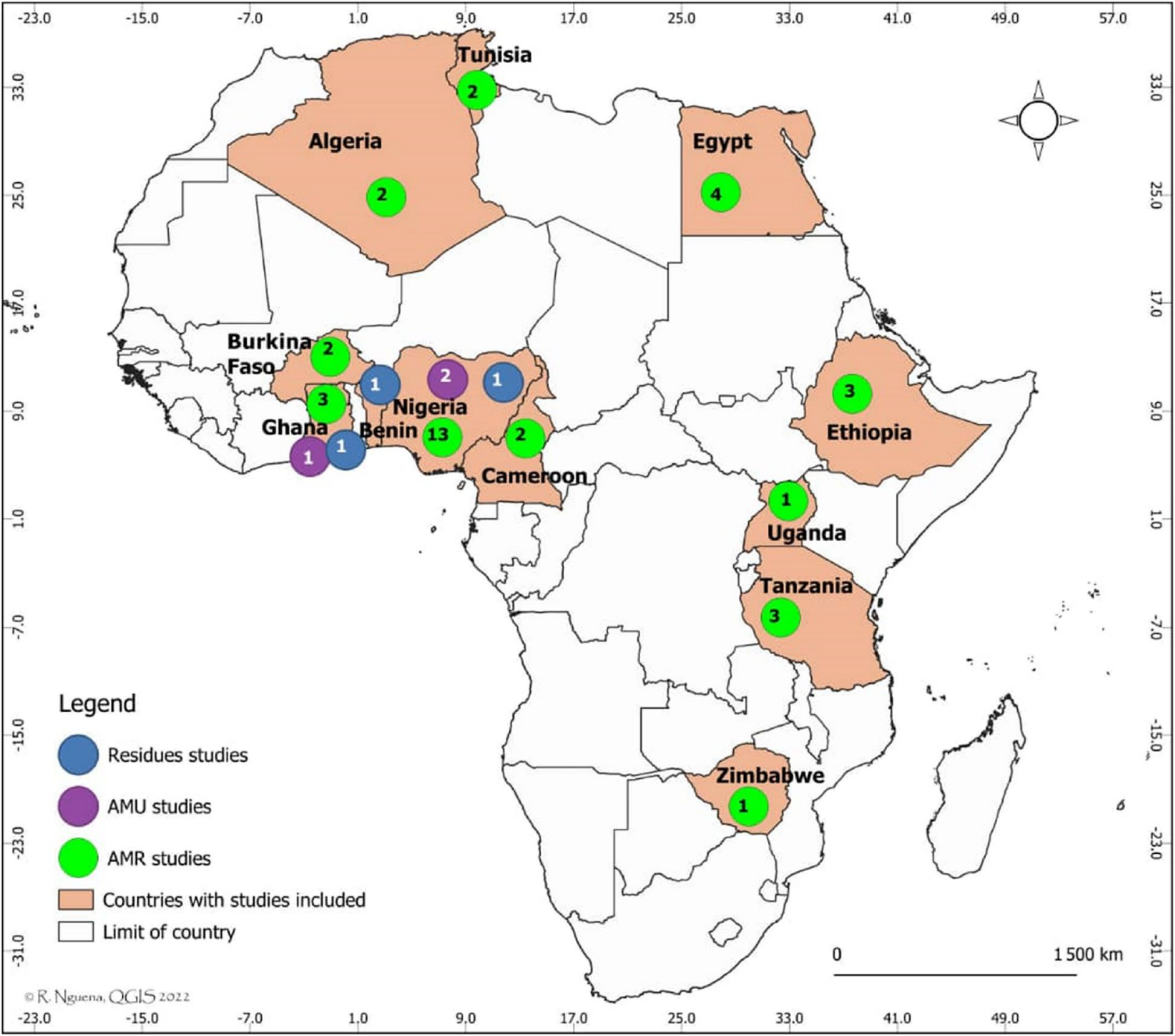
Map of Africa showing the study sites and the number of articles included in the review and meta-analysis

A total of 113 citations were identified using an online database search strategy in this study. Forty duplicate papers were removed, and 73 records were screened for eligibility based on a review of the title and abstract content. Twelve papers were excluded because they were not relevant to the research objectives. Of the 61 full-text articles assessed for eligibility, 41 met the inclusion criteria and were retained for analysis (Fig. 1). Three (03) studies reported the outcomes of AMU in aquaculture [5, 21, 22], and three (03) studies investigated ARs in fish [21, 23, 24]. A total of 35 studies reported the outcome of AMR in aquaculture [6, 23, 25–57]. The studied articles were journal papers (100%), published in English (100%), and included cross-sectional perspective (32; 91.4%) and longitudinal studies (3; 8.6%), with the majority originating from West Africa (23; 56.1%) and North Africa (8; 19.5%) (Fig. 2). Most of the articles on AMR originated from Nigeria (13; 37.1%), Egypt (4; 11.4%), Ethiopia (3; 8.6%) and Tanzania (3; 8.6%) and focused on fresh fish (91.4%), with *Oreochromis niloticus* (13; 59.4%), *Clarias grariepinus* (11; 34.4%), and *Sardina pilchardus* (2; 6.3%) being the most represented species. Samples were commonly collected from markets (14; 40.0%), fish farms (13; 37.1%), and natural milieu (8; 22.9%). With regard to the type of sample, the gut (8; 22.9%), muscle (8; 22.9%), gill (7; 20.0%), gut content (5; 14.3%) and skin (3; 8.6%) were the most common pathogens identified.

Overall, 24 types of pathogens were isolated and tested against 62 different antimicrobial agents, 42 of which were critically important antimicrobial agents [β-lactams (17; 27.4%), cephalosporin (9; 14.5%), quinolone (6; 9.7%), macrolide (4; 6.45%), and carbapenem (2; 3.2%)]. Additionally, 20 were classified as important antimicrobial agents [aminoglycoside (8; 12.90%), sulfonamide (5; 8.06%), phenicol (3; 4.84%), and tetracycline (3; 4.84%)]. The bacteria most commonly reported in the articles were *Escherichia coli* (16; 45.7%), *Salmonella* spp. (10; 28.6%), *Staphylococcus* spp. (8; 22.9%), *Aeromonas* spp. (8; 22.9%), *Proteus* spp. (8; 22.9%), *Klebsiella* spp. (8; 22.9%), and *Enterobacter* spp. (8; 22.9%) (Table 1).

**Table 1:** Distribution and characteristics of the studies included in the review and meta-analysis of antimicrobial use, residues and antimicrobial resistance in fish and other aquatic sectors in Africa

Factors	Number of studies on AMR (*n*=35)	Number of studies on AMU (*n*=3)	Number of studies on residue (*n*=3)	References
Country				
Algeria	2 (5.7%)			[54, 57]
Benin			1 (33.3%)	[24]
Burkina-Faso	2 (5.7%)			[48, 49]
Cameroon	2 (5.7%)			[45, 51]
Egypt	4 (11.4%)			[6, 30, 40, 43]
Ethiopia	3 (8.6%)			[33, 34]
Ghana	2 (5.7%)	1 (33.3%)	1 (33.3%)	[23, 37]
Nigeria	13 (37.1%)		1 (33.3%)	[26, 29, 31, 35, 39, 41, 42, 44, 52, 53, 56]
Tanzania	3 (8.6%)	2 (66.7%)		[27, 38]
Tunisia	2 (5.7%)			[46, 55]
Uganda	1 (2.9%)			[50]
Zimbabwe	1 (2.9%)			[36]
Study design				
Cross sectional study	32 (91.4%)	3 (100%)	3 (100%)	
Longitudinal study	3 (8.6%)			
Aquaculture product study				
Fresh fish	32 (91.4%)			
Shrimp	4 (11.4%)			
Dry fish	1 (2.9%)			
Fish species				
*Oreochromis niloticus*	13(59.4%),			
*Clarias gariepinus*	11(34.4%),			
*Sardina pilchardus*	2(6.3%)			
*Mugil cephalus*	1(3.2%)			
*Sarp sarpa*	1(3.2%)			
*pagellus acarne*	1(3.2%)			
*Engraulis encrasicolus*	1(3.2%)			
*Boops boops*	1(3.2%)			
*Trachurus trachurus*	1(3.2%)			
Origin of aquatic product				
Natural milieu	8 (22.9%)			
Fishmongers (Fish market)	14 (40.0%)			
Fish farm	13 (37.1%)			
Consumer	2 (5.7%)			
Ornamental fish	1 (2.9%)			
Fisherman	3 (8.6%)			
Study Sample				
Gills	7 (20.0%)			
Gut	8 (22.9%)			
Gut content	5 (14.3%)			
Ascetic fluid	1 (2.9%)			
Gills, intestine and skin mixture	1 (2.9%)			
Brain	1 (2.9%)			
Liver, spleen and kidney mixture	1 (2.9%)			
kidney,	1 (2.9%)			
Liver	1 (2.9%)			
Spleen	1 (2.9%)			
Muscle	8 (22.9%)			
Dry fish	1 (2.9%)			
Whole fish swab	2 (5.7%)			
Working knife and cutting board swab	1 (2.9%)			
Ready to eat fish	1 (2.9%)			
Workers hand swab	1 (2.9%)			
Fish container swab	1 (2.9%)			
Swab from skin gill and other relevant body part	1 (2.9%)			
Liver, gills and kidney swab	1 (2.9%)			
Head, middle and tail region	2 (5.7%)			
Gills and stomach mixture	1 (2.9%)			
Head kidney, liver, spleen and brain	1 (2.9%)			
Skin	3 (8.6%)			
Intestine and gills (mixture)	1 (2.9%)			
Bacteria isolates in studies				
*Escherichia coli*	16 (45.7%)			[6, 23, 26, 27, 31–34, 36, 37, 39, 44, 47, 54, 56, 57],
*Klebsiella spp*	8 (22.9%)			[6, 23, 36, 37, 39, 47, 54, 56]
*Enterobacter *spp	8 (22.9%)			[6, 23, 29, 36, 47, 54, 56, 57]
*Proteus *spp	8 (22.9%)			[23, 27, 36, 37, 39, 47, 54, 57]
*Citrobacter *spp	3(8.6%)			[37, 41]
*Enteriobacteriaceae*	2 (5.7%)			[25, 29, 29, 36, 48, 50]
*Vibrio *spp	4 (11.4%)			[23, 26, 35, 37, 41, 44, 47, 49, 56, 57]
*Salmonella *spp	10 (28.6%)			[30, 36, 39, 45, 46, 50, 52, 53]
*Aeromonas *spp	8 (22.9%)			[26, 27, 36, 39, 41, 42, 44, 50, 51, 51, 56]
*Bacillus *spp	2 (5.7%)			[26, 27, 39, 39]
*Plesiomonas shigelloides*	2 (5.7%)			[43]
*Staphylococcus *spp	8 (22.9%)			[37, 43, 56, 57]
*Enterococcus *spp	2 (5.7%)			[50, 51]
*Streptococcus *spp	3 (8.6%)			[23, 39]
*Lactococcus garvieae*	1 (2.9%)			[29, 41, 44, 50, 51]
*Aerococcus viridans*	1 (2.9%)			[51]
*Serratia *spp	4 (11.4%)			
*Edwarsiella tarda*	2 (5.7%)			
*Shigella *spp	2 (5.7%)			
*Pseudomonas *spp	5 (14.3%)			
*Acinobacter *spp	1 (2.9%)			
Antimicrobial agent tested in studies (*n*=62)				
Cephalosporin	9 (14.5%)			
Quinolones	6 (9.7%)			
Glycopeptides	1 (1.6%)			
Macrolide	4 (6.5%)			
Aminoglycosides	8 (12.9%)			
Polymixin	1 (1.6%)			
Carbapenem	2 (3.2%)			
β-lactams	17 (27.4%)			
Sulfonamides	5 (8.1%)			
Phenicols	3 (4.8%)			
Phenicols	3 (4.8%)			
Sulfonamides-trimethoprim	3 (4.8%)			

*AMU *antimicrobial use, *AMR *antimicrobial resistance

### Antimicrobial use in aquaculture

Of the three articles that reported the outcomes of AMU in aquaculture, two were from Nigeria and one was from Ghana (Table 1). Tetracyclines (3/3) and penicillin (2/3) were the most common antimicrobial agents reported in these studies, followed by sulfamethoxazole, virginiomycin, erythromycin, enrofloxacin, and chloramphenicol (1/3). One article reported the frequency of AMU and the indication for usage. Agoba et al.[5] reported that two out of nine hatcheries investigated in Ghana used tetracycline or chloramphenicol in fish feed. Olatoye and Basiru [22] reported that 90% of the 20 fish farmers investigated in their study used oxytetracycline, penicillin, and enrofloxacin for preventive measures, treatment, and growth promotion. Alarape and Adelewo reported that oxytetracycline (69.8%), penicillin (25%), erythromycin (25%), and enrofloxacin (22.4%) were more commonly used in fish farms than were sulfamethazole (12.1%) and virginiomycin (6%) in 116 fish farms in Nigeria [21].

### Antimicrobial residues in fish

Of the three articles that reported the outcomes of ARs in fish products, two focused on qualitative analysis of the presence of tetracycline, amphenicols, and beta-lactams, and one focused on the quantitative analysis of tetracycline. Out of a total of 144 samples of *Clarias gariepinus* and *Oreochromis niloticus* screened in Benin, a prevalence of 11.1% of tetracycline residue was reported. The residue was more prevalent in *Clarias gariepinus* (16.7%) than in *Oreochromis niloticus* (5.6%) [24]. Donkor et al. [23] examined 100 samples of tilapia (*Oreochromis niloticus*) gills from the Ghana market and reported an overall prevalence of 7% AR in fish. Among the 60 muscle samples of fresh and smoked *Clarias gariepinus* strains analyzed in Nigeria, 56.7% of the total tetracycline residue was detected. In addition, the reported concentration of 236 ng/g was higher than the recommended maximum residue level (MRL) of 200 ng/g [21].

### Antimicrobial resistance in aquaculture products

**Fig. 3:**
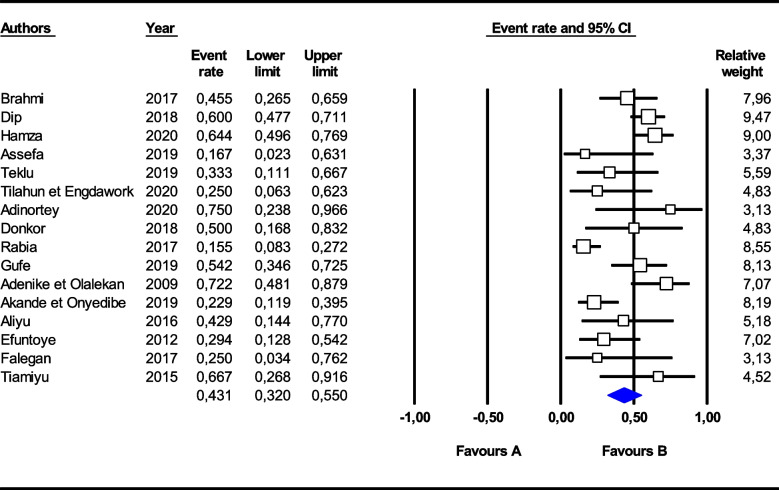
Forest plot of the pooled prevalence of multidrug-resistant *Escherichia coli* in fish farming in Africa

Of the 35 studies that reported outcomes of AMR in aquaculture, 29 were assessed as high quality with AMR prevalence data and were included in the meta-analysis. Higher levels of resistance of *Escherichia coli* were detected for ampicillin (87.1%) [95% CI (62.8–96.4)], cotrimoxazole (65.1%) [95% CI (38.0–85.1)] and tetracycline (66.4%) [95% CI (46.3–81.8]) than for ceftriaxone (15.0%) [95% CI (3.6–45.2)], ciprofloxacin (15.1%) [95% CI (5.8–33.7)] and gentamicin (18.0%) [95% CI (7.9–36.1)]. Overall, an *Escherichia coli* multidrug resistance rate of 43.1% [95% CI (32.0–55.0)], I^2^ = 69.5%, *p* < 0.001] was observed (Fig. 3).

**Fig. 4:**
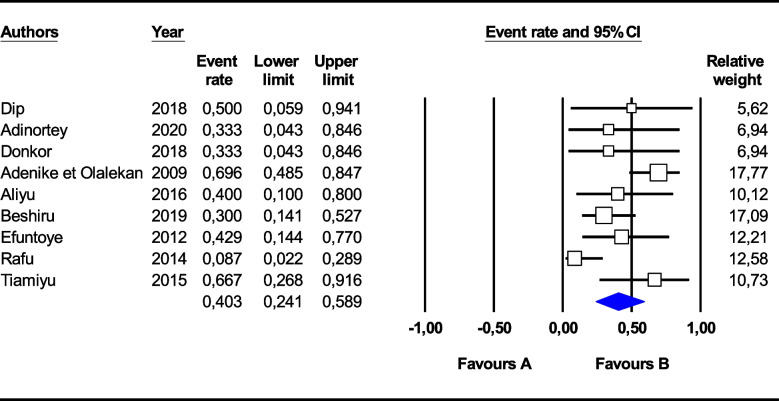
Forest plot of the pooled prevalence of *Salmonella* spp. multidrug resistance in fish farming in Africa

Concerning *Salmonella* spp., a higher pooled resistance rate was observed for amoxicillin (74.9%, 95% CI 39.6–93.1) and cotrimoxazole (68.9%, 95% CI 30.3–91.9) than for cefotaxime (7.4%, 95% CI 0.9–42.1), ciprofloxacin (7.8%, 95% CI 1.7–29.6), chloramphenicol (11.3%, 95% CI 3.0–34.2), and gentamicin (17.3%, 95% CI 5.0–45.6). Overall, a *Salmonella* spp. multidrug resistance rate of 40.3% [95% CI (24.1–58.1)] (I^2^ = 52.09%, *p* < 0.03) was observed (Fig. 4).

**Fig. 5:**
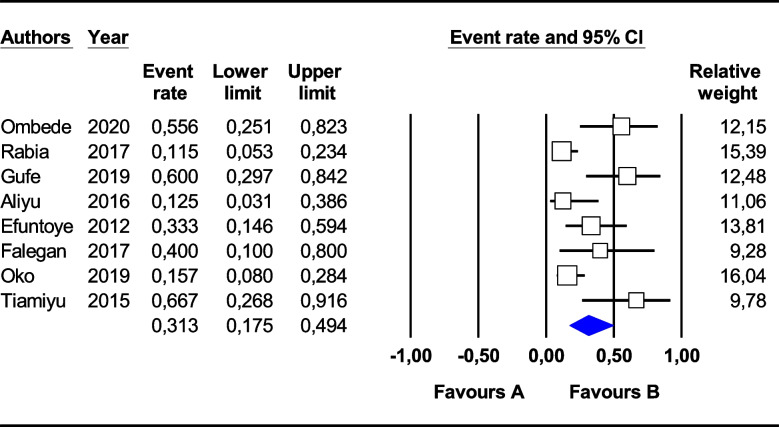
Forest plot of the pooled prevalence of *Staphylococcus* spp. multidrug resistance in fish farming in Africa

For *Staphylococcus* spp., a higher pooled resistance rate was observed for ampicillin (45.6%; 95% CI (11.2–84.8)) and tetracycline (37.5%; 95% CI (18.2–61.8) than for gentamycin (8.9%; 95% CI (1.9–33.3)), nitrofurantoin (11.0%; 95% CI (2.7–35.7]), ciprofloxacin (15.7%; 95% CI (3.9–46.2)) and erythromycin (27.8%; 95% CI (10.4–56.2)) (Table 2). Overall, a *Staphylococcus* spp. multidrug resistance rate of 31.3% [95% CI (17.5–49.4)], I^2^ = 69.46%, *p* < 0.002 was observed (Fig. 5). For the African subregion where the studies were reported, the pooled prevalence of MDR *Escherichia coli* was significantly (*p* < 0.05) lower in East Africa than in North and West Africa (Table 3).

**Table 2:** Validity analysis of the scale to assess post-stroke depression with any depression

Bacteria reported in studies	Antimicrobial agents	Number of studies	Pooled prevalence of AMR (95% IC)
*Escherichia coli*	**Beta-lactams**		
	Ampicillin	10	87.1 (62.8-96.4)
	Ceftriaxon	4	15.0 (3.6-45.2)
	Cefotaxim	5	67.1 (30.3-90.6)
	Cefuroxim	4	65.9 (7.8-97.8)
	Ceftazidime	4	32.0 (5.3-79.8)
	**Quinolones**		
	Nalidixic acid	5	49.1 (15.7-83.4)
	Ofloxacin	4	35.2 (9.1-74.8)
	Ciprofloxacin	10	15.1 (5.8 -33.7)
	**Nitrofuran**		
	Nitrofurantoin	6	21.6 (12.1-35.5)
	**Sulfonamides-trimethoprim**		
	Cotrimoxazole	5	65.1 (38.0-85.1)
	**Aminiglycosides**		
	Gentamicin	15	18.0 (7.9-36.1)
	Streptomycin	4	36.4 (13.5-67.7)
	**Tetracyclines**		
	Tetracycline	10	66.4 (46.3-81.8)
	**Phenicols**		
	Chloramphenicol	4	44.4 (13.4-80.6)
*Aeromonas* spp	**Beta-lactams**		
	Ampicillin	7	91.8 (73.4-97.8)
	Ceftazidime	4	32.9 (11.0-66.1)
	**Aminoglycosides**		
	Gentamicin	7	7.7 (1.8-27.5)
	Streptomycin	5	23.4 (8.8-49.2)
	**Sulfonamides-trimethoprim**		
	Trimethoprim-sulfamethoxazole	5	23.4 (5.9-59.7)
	**Tetracyclines**		
	Tetracycline	5	49.7 (35.9-63.6)
*Citrobacter* spp	**Aminoglycosides**		
	Gentamicin	4	15.5 (1.7-66.3)
*Enterobacter* spp	**Tetracyclines**		
	Tetracycline	4	57.4 (18.8-88.7)
*Klebsiella* Spp	**Beta-lactams**		
	Ampicillin	4	86.4 (60.1-96.4)
	Cefotaxime	5	65.9 (45.6-81.7)
	**Quinolones**		
	Ciprofloxacin	4	29.6 (11.9-56.8)
	**Sulfonamides-trimethoprim**		
	Cotrimoxazole	4	55.9 (32.8-76.7)
	**Aminoglycosides**		
	Gentamicin	7	33.8 (15.8-55.1)
	**Tetracyclines**		
	Tetracycline	5	81.5 (66.7-90.6)
*Proteus* spp	**Quinolones**		
	Ciprofloxacin	4	20.9 (2.2-75.8)
	**Aminoglycosides**		
	Gentamicin	4	49.5 (16.3-83.2)
*Pseudomonas* spp	**Aminoglycosides**		
	Gentamicin	4	41.1 (13.7-77.5)
*Salmonella* spp	**Beta-lactams**		
	Amoxicillin	5	74.9 (39.6-93.1)
	Ampicillin	8	50.7 (17.4-83.3)
	Cefotaxime	5	7.4 (0.9-42.1)
	**Phenicols**		
	Chloramphenicol	7	11.3 (3.0-34.2)
	**Aminoglycosides**		
	Gentamicin	10	17.3 (5.0-45.6)
	Streptomycin	4	35.5 (14.8-63.5)
	**Quinolones**		
	Ofloxacin	4	29.2 (0.8-66.2)
	Nalidixic acid	5	18.1 (2.8-62.5)
	Ciprofloxacin	6	7.8 (1.7-29.6)
	**Nitrofuran**		
	Nitrofurantoin	4	41.5 (15.9-72.7)
	**Sulfonamides-trimethoprim**		
	Cotrimoxazole	4	68.9 (30.3-91.9)
	**Tetracycline**		
	Tetracycline	10	40.3 (19.5-65.4)
*Staphylococcus* spp	**Beta-lactams**		
	Ampicillin	4	45.6 (11.2-84.8)
	**Quinolones**		
	Ciprofloxacin	6	15.7 (3.9-46.2)
	**Macrolide**		
	Erythromycine	6	27.8 (10.4-56.2)
	**Aminoglycosides**		
	Gentamicin	6	8.9 (1.9-33.3)
	**Nitrofuran**		
	Nitrofurantoin	5	11.0 (2.7-35.7)
	**Sulfonamides-trimethoprim**		
	Trimethoprim-Sulfamethazole	4	6.8 (1.5-25.2)
	**Tetracycline**		
	Tetracycline	6	37.5 (18.2-61.8)
*Vibrio* spp	**Beta-lactams**		
	Ampicillin	4	56.7 (17.3-89.1)
	**Aminoglycosides**		
	Streptomycin	4	17.0 (4.6-46.5)

**Table 3:** Pooled prevalence of multidrug resistance in more distributed bacteria based on a meta-analysis of studies with respect to the African subregion

Bacteria more distributed	Study area	Number of studies	Pooled prevalence of MDR (95% CI)	*p* value
*Escherichia coli*	North Africa	3	59.5 (50.8–67.6)	
	West Africa	8	45.6 (29.5–62.6)	0.033
	East Africa	4	20.2 (12.7–30.6)	
*Enterobacter* spp	North Africa	2	54.4 (6.8–95.2)	0.325
	West Africa	3	36.6 (16.1–63.3)	
*Aeromonas* spp	North Africa	3	31.1 (21.3–43.5)	0.640
	West Africa	2	58.9 (24.0–86.7)	

## Discussion

Despite the decreasing use of antimicrobial agents in recent decades, partly due to the ban on growth promoting treatments in many high-income countries (Sweeden, South Korea, the USA, Canada, Mexico, Japan, and China) [58], information on AMU in fish farming in low- and middle-income countries is scarce, hindering the assessment of human, animal, and environmental risks. This study was performed to summarize published data on AMU, ARs and AMR in aquaculture in Africa as key elements for decision making and policies. At least 27% of fish farmers use antimicrobials for disease prevention and control. Tetracycline was identified as the common class of antimicrobial used in fish farms across the African region [5, 21, 22]. Oxytetracycline is known to be a common antimicrobial agent used in fish farms, especially in hatcheries [59–62]. The systematic use of tetracycline could be explained by its broad-spectrum activity against furunculsis, Vibrio [63], ulcer disease, Pseudomonas disease, and bacterial hemorrhagic septicaemia [64]. In addition, tetracycline is cheaper and more readily available than other alternative drugs used in aquaculture [65]. Penicillin, erythromycin, enrofloxacin, and sulfamethazole were reported to be used in fish farms in Nigeria. These antimicrobial agents, classified as the highest priority critically important antimicrobial agents or highly important antimicrobial agents by the World Health Organization, highlight the urgent need for antimicrobial regulation, reinforcement, control and reporting in aquaculture [66]. Other consequences of the use of antimicrobial agents in fish farms include the deposition of residues in muscles designated for human consumption irrespective of the route or purpose of administration before they are completely metabolized or excreted from the body [67]. The presence of residues in fish could pose a public health risk to consumers [22]. The prevalence of residue in fish in Africa was higher than the 1% reported in European countries[24, 68]. The main reasons include poor drug regulation in animals, a lack of complete monitoring from prescription to antimicrobial agent use, a lack of updated AMU and treatment guidelines in most African countries[65], the use of noncompliant (substandard drugs with lower concentrations of active ingredients than those stated on labels) veterinary drugs[69], and detection methods that are often inadequate or unavailable at all to comply with limit values and the absence of certification systems regarding food products of animal origin[68].

Tetracyclines, β-lactams (penicillin) and phenicols (chloramphenicol) were mostly detected in fish. Particular attention should be given to antimicrobial agents that are toxic to humans even at low concentrations, such as chloramphenicol and tetracycline. Various studies have shown that ARs from food can negatively impact human health through allergic reactions, mutations in cells, imbalances in the intestinal microbiome, and ultimately, the presence of multiresistant microorganisms [68]. Evidence studies have reported that chloramphenicol residues may be associated with hematological disorders, including aplastic anemia in humans, while sulfametazine, oxytetracycline and furazolidone may induce carcinogenicity[70]. This inability to set the threshold value and the shortcomings of the dossier led to its classification as a substance prohibited for use in food-producing animals[65]. A high concentration of tetracycline residue (236 ng/kg) in fish and products that exceed the allowable residue limits (200 ng/kg) [71] poses a serious threat to public health. Heat treatments that occur during cooking can reduce the risk of ingesting tetracyclines but do not guarantee the breakdown of these antimicrobial residues in animal products, such as broiler meat [72]. The high stability of β-lactams represents a significant risk to human health because the residues of these antimicrobial agents can remain in foodstuff after heat treatment and, therefore, can reach the dairy industry and consumers [73].

In the present study, 35 articles reported the outcome of AMR in various bacteria in Africa. Enterobacterales isolated from aquaculture products, such as *Escherichia coli and Klebsiella* spp., show a high rate of antimicrobial resistance to ampicillin, cefotaxim, cotrimoxazole and tetracycline, while *Salmonella* spp. exhibit a high rate of antimicrobial resistance to amoxicillin, ampicillin, and cotrimoxazole. These antimicrobial agents are the most inexpensive broad-spectrum drugs and are therefore frequently used [65]. The use of antimicrobial agents with a broader spectrum affects a greater number of bacterial taxa and may increase the risk of selecting bacteria harboring resistance genes compared with agents with a narrower spectrum. In addition, it may increase the risk of suppressing and eliminating susceptible commensal microbiota, which generally outcompete resistant strains [26]. Approximately 80% of antimicrobials administered through feeds to aquatic farmed animals disseminate to nearby environments (water and sediment), where they remain active for months at concentrations allowing selective pressure on bacterial communities and favoring AMR development [74]. Additionally, manure from treated animals [75], human feces and urine [76] are indirect sources of antimicrobial agents and their residues in aquaculture [75, 76]. Independent of these practices, the aquatic environment is considered the major pool for antimicrobial agents accumulated from effluent discharged after treatment, and surface runoff has the same undesired effect on the sensitivity of aquatic pathogens to antimicrobial agents [77]. In this review, the high rate of resistance to multiple classes of antimicrobial agents in aquatic products raises the urgent question of the therapeutic efficacy of first-line antimicrobial agents and the degradation of last-resort therapeutics during serious infections due to multiresistant bacteria [78].

The high MDR prevalence observed in *Enterobacter* spp. and *Escherichia coli* emphasizes the importance of Enterobacteriaceae in aquatic environments as carriers of AMR genes and determinants of virulence. Hence, there is a need for in-depth monitoring of aquatic environments as a source of the emergence and spread of AMR [54]. This review highlights serious concerns relating to the use of ampicillin, tetracycline and cotrimoxazole as antimicrobial agents of choice for optimal therapy of common pathogens and the difficulty of treating Enterobacteriaceae disease in Africa. Although this study is based on the state of knowledge on AMU, ARs, and AMR in aquaculture on the African continent, it suffers from a lack of data concerning AMU and residue in aquaculture. However, the few existing data on AMU are exclusively focused on the percentages of farms using antimicrobial agents rather than on defined daily doses, as recommended by the World Health Organization. Additionally, the majority of studies on AMR have not provided an understanding of the dynamics of resistance transmission because these studies are interested in phenotypic rather than molecular aspects. This review highlights the need for the implementation of AMR surveillance based on one health approach to develop surveillance strategies at the level of each African country. Thus, as suggested by Gazal et al. [78], each state would begin by enforcing the complete restriction of the use of medically important antimicrobial agents for the prevention of pathologies in aquaculture or as growth promoters. The prudent use of antimicrobial agents under veterinary control must be the other line of action to ensure the safety of aquatic products.

## Conclusion

The present review highlighted the general lack of information about AMR surveillance in aquaculture, especially concerning AMU and residue. The high prevalence of resistance to the most commonly used antimicrobial agents and the level of MDR bacteria imposed by certain isolated bacteria reveal the real threat posed by AMR to public health. Furthermore, Africa could benefit from developing strategies to increase awareness and understanding of the AMR problem through effective communication, education and training; optimizing the use of antimicrobial agents; reducing the incidence of infection through effective sanitation, hygiene, and implementation of good farm biosecurity practices and prevention measures; and above all, strengthening knowledge through surveillance and research.

## Data Availability

The data sets used and/or analyzed during the current study are available from the corresponding author upon reasonable request.
